# Mitochondrial DNA Haplogroups and Breast Cancer Risk Factors in the Avon Longitudinal Study of Parents and Children (ALSPAC)

**DOI:** 10.3390/genes9080395

**Published:** 2018-08-01

**Authors:** Vivienne Riley, A Mesut Erzurumluoglu, Santiago Rodriguez, Carolina Bonilla

**Affiliations:** 1 MSc Genomic Medicine Programme, G7, College House, St Luke’s Campus University of Exeter, Exeter, Devon EX2 4TE, UK; vivienne.riley@gmail.com; 2 Genetic Epidemiology Group, Department of Health Sciences, University of Leicester, Leicester LE1 7RH, UK; ame26@leicester.ac.uk; 3 MRC Integrative Epidemiology Unit, Population Health Sciences, Bristol Medical School, University of Bristol, Oakfield House, Oakfield Grove, Bristol BS8 2BN, UK; Santi.Rodriguez@bristol.ac.uk; 4 Integrative Cancer Epidemiology Program, Population Health Sciences, Bristol Medical School, University of Bristol, Oakfield House, Oakfield Grove, Bristol BS8 2BN, UK; 5 Departamento de Medicina Preventiva, Faculdade de Medicina, Universidade de São Paulo, São Paulo 01246-903, Brazil

**Keywords:** mitochondrial DNA, haplogroups, breast cancer, risk factors, ALSPAC, Europeans

## Abstract

The relationship between mitochondrial DNA (mtDNA) and breast cancer has been frequently examined, particularly in European populations. However, studies reporting associations between mtDNA haplogroups and breast cancer risk have had a few shortcomings including small sample sizes, failure to account for population stratification and performing inadequate statistical tests. In this study we investigated the association of mtDNA haplogroups of European origin with several breast cancer risk factors in mothers and children of the Avon Longitudinal Study of Parents and Children (ALSPAC), a birth cohort that enrolled over 14,000 pregnant women in the Southwest region of the UK. Risk factor data were obtained from questionnaires, clinic visits and blood measurements. Information on over 40 independent breast cancer risk factor-related variables was available for up to 7781 mothers and children with mtDNA haplogroup data in ALSPAC. Linear and logistic regression models adjusted for age, sex and population stratification principal components were evaluated. After correction for multiple testing we found no evidence of association of European mtDNA haplogroups with any of the breast cancer risk factors analysed. Mitochondrial DNA haplogroups are unlikely to underlie susceptibility to breast cancer that occurs via the risk factors examined in this study of a population of European ancestry.

## 1. Introduction

The relationship between mitochondrial DNA (mtDNA) and breast cancer has been frequently explored. Carcinogenesis has been associated with oxidative stress, with mitochondria acting as a major source of production of reactive oxygen species (ROS) [1]. Additionally, the lack of protective histones and a limited capacity for DNA repair [2,3] has meant that the mitochondrial genome is particularly susceptible to damage by ROS, which in turn could affect the mitochondrial role in energy metabolism, apoptosis and aging [4]. Most publications have focused on somatic mutations in mtDNA. However, germline variants that subtly affect mitochondrial functioning may also lead to a build-up of ROS, resulting in an elevated cancer risk [4], and their study has thus become more frequent [5].

Since mtDNA is maternally inherited, it has been suggested that it could underlie the observation that having a mother diagnosed with breast cancer increases a woman’s risk of the disease.

During evolution mtDNA mutations have segregated and clustered in groups of related haplotypes or haplogroups (also referred to as clades) that differ prominently in frequencies across continents [6,7]. The geographic patterning of mtDNA lineages was attributed to founder effects although natural selection has been recently postulated as a more probable cause. Adaptation to climate and nutrition may have been the environmental selective factors to drive clade differences by region given that haplogroups exhibit diverse metabolic capacities [6,8]. Thus, lineages that are advantageous in a particular environment could become maladaptive when the environment changes contributing to the development of disease.

Nine major mtDNA haplogroups have been identified in Europeans (namely H, I, J, K, T, U, V, W, X). Haplogroup H is the most frequent, though the frequencies of individual clades vary within Europe [9].

The association of mtDNA variants and haplogroups with cancer and other diseases has been extensively explored in the European population. For instance, there are reports on breast cancer [10,11], prostate cancer [12], meningococcal disease [13], neurological diseases [14], type 2 diabetes mellitus [15], infertility [16], obesity [17], acquired immunodeficiency syndrome (AIDS) progression [18], stroke [19], and osteoarthritis [20]. Results have been inconsistent across studies of the same disease. This has been attributed to a number of problems relating to study design and data analysis, such as population stratification, genotyping error, small sample sizes or inadequate statistical approaches [21,22,23,24]. The case of breast cancer studies is particularly noteworthy [23].

In order to elucidate the putative role of mtDNA in breast cancer we investigated the distribution of European mtDNA haplogroups across a range of well-known and possible risk factors for breast cancer in mothers and children of the Avon Longitudinal Study of Parents and Children (ALSPAC). If mtDNA variation plays a role in breast cancer susceptibility it may do so via risk factors that lie in the causal pathway to disease. Thus, we might be able to detect an association of mtDNA haplogroups with these exposures with less bias in a large and substantially homogeneous cohort like ALSPAC.

## 2. Materials and Methods

### 2.1. Avon Longitudinal Study of Parents and Children (ALSPAC)

The Avon Longitudinal Study of Parents and Children (ALSPAC) is a prospective, population-based birth cohort study that recruited 14,541 pregnant women residing in Avon County, United Kingdom, with an expected delivery date between 1 April 1991 and 31 December 1992. From these initial pregnancies, there were a total of 14,676 fetuses, resulting in 14,062 live births and 13,988 children who were alive at one year of age. When the oldest children were approximately seven years of age, an attempt was made to bolster the initial sample with eligible cases who had failed to join the study originally. The total sample size for analyses using any data collected after the age of seven is therefore 15,247 pregnancies, resulting in 15,458 fetuses, of which 14,775 were live births and 14,701 were alive at one year of age. Data were gathered through self-completed questionnaires or assessment at research clinics at regular intervals. The study is described in detail elsewhere [25,26] (http://www.bristol.ac.uk/alspac/). Hormone level measurements (i.e., circulating sex hormones, insulin growth factors and insulin growth factor binding proteins) were reported earlier [27,28,29,30,31]. Please note that the study website contains details of all the data that are available through a fully searchable data dictionary: http://www.bris.ac.uk/alspac/researchers/data-access/data-dictionary/.

Ethical approval for the study was obtained from the ALSPAC Ethics and Law Committee and the Local Research Ethics Committees (http://www.bristol.ac.uk/alspac/researchers/research-ethics/). Written informed consent was obtained from all participants in the study.

### 2.2. Breast Cancer Risk Factors

The selection of breast cancer risk factors was based on worldwide research data published by the World Cancer Research Fund [32] and Cancer Research UK [33]. 

Lifestyle factors showing robust evidence for increasing the risk of breast cancer have been identified in premenopausal women, postmenopausal women, or both. Risk factors for postmenopausal breast cancer are: overweight or obesity and a greater weight gain in adulthood. Having a greater birth weight is a risk factor for premenopausal breast cancer. Alcohol intake and a greater linear growth (marked by adult attained height) have been associated with both pre- and postmenopausal breast cancer. Conversely, factors that reduce the risk of breast cancer include increased physical activity, and breastfeeding, whereas being overweight or obese acts as a protective factor in premenopausal women.

Other established risk factors are older age, White ethnicity, family history of breast cancer, a prior diagnosis of cancer, early menarche, late natural menopause, nulliparity, first pregnancy after the age of 30, hormone replacement therapy, use of oral contraceptives, high bone mineral density, diabetes mellitus and exposure to radiation such as X-rays.

Possible breast cancer risk factors include smoking and night shift work. In contrast, a healthy diet and regularly taking aspirin or other non-steroidal anti-inflammatory drugs are considered protective factors.

We also tested women having had reproductive surgery (hysterectomy and/or oophorectomy), and using non-oral hormonal contraceptives, which may affect breast cancer risk by altering hormone concentrations, and serum levels of sex hormone binding globulin (SHBG), testosterone, androstenedione, dehydroepiandrosterone-sulfate (DHEAS), insulin like growth factor I (IGF-I), insulin-like growth factor II (IGF-II) and insulin-like growth factor binding protein 3 (IGFBP-3).

More detailed information on the association of the risk factors considered with breast cancer can be found in the literature [34,35,36,37,38].

Data on most risk and protective factors were available in ALSPAC from mothers, children or both.

### 2.3. Variables Analyzed

Details on the continuous and categorical variables examined in mothers and children are given in Tables S1 and S2 showing the complete set of ALSPAC participants of European ancestry. Data were obtained from questionnaires answered by the subject or by the subject’s mother during childhood, as well as from clinic visits at different time points. We limited the analysis to variables measured approximately every two years in the children.

In some cases, usually when numbers were low, we created new variables that reflected whether the individual had ever experienced the activity or shown the trait of interest by combining the different instances where it was assessed. We did this for smoking, having diabetes, having had cancer, having undergone a hysterectomy and/or an oophorectomy, doing night shift work, taking oral contraceptives, using non-oral hormonal contraceptives, receiving hormone replacement therapy, taking aspirin, being a biological parent, undertaking physical activity and getting X-rays. The variable age of menarche in the children was determined by integrating all the information provided by the mother and the child [39].

### 2.4. Mitochondrial DNA Genotyping

Genotyping methods for mitochondrial and nuclear DNA polymorphisms in ALSPAC have been previously described [40]. Haplogroup assignment was performed using HaploGrep [41]. Mitochondrial DNA haplogroups of mothers were inferred from those of their children in view of the maternal inheritance of mtDNA.

Samples with a quality score of more than 80% were included in the analysis (238 samples were excluded, Table S3). Additionally, we ran the analysis using a quality score cut-off point of >90%.

We grouped the clades as follows: European = H (H + V + subclade R0), J, K, T, U, other European (I + W + X + subclades N1, R1, R3); South Asian = M + subclade R5; East/Southeast Asian = A + C + D; and African = L. The ‘other European’ group consisted of haplogroups that were present in less than 3% of the sample.

We restricted our analysis to all individuals who were of European genomic ancestry (as detected by a multidimensional scaling analysis seeded with haplotype map (HapMap)2 individuals) or self-identified as White (if data on genomic ancestry was missing) and carried a European mtDNA haplogroup. There was information on 7781 mtDNA haplogroups in our working dataset.

### 2.5. Statistical Analysis

We used linear regression to investigate the association of continuous variables with mtDNA haplogroups and chi-squared tests to examine differences in categorical variables. Adjustment for confounders was carried out using linear and logistic regression models with continuous and categorical variables, respectively. When categorical variables exhibited more than two ordered categories ordinal logistic regression was run, also adjusted for confounders. Confounders introduced in the models were age, sex, gestational age and the top 10 principal components accounting for population stratification, where appropriate.

We checked that the residuals of the linear regression of each continuous variable on mtDNA haplogroups were normally distributed. Only residuals for DHEAS levels in children showed a markedly non-normal distribution, and therefore the variable was natural log-transformed [42].

Similarly to Howe et al. [40], we used pairwise correlation to determine the number of independent variables to account for when applying the Bonferroni correction for multiple testing [43]. Polychoric correlation was used with binary and ordinal variables. Variables showing a correlation coefficient of 0.8 and above were considered non-independent (data not shown). There were 43 independent variables (out of 59) in the mothers and 48 independent variables (out of 86) in the children, therefore the multiple testing adjusted *p*-value cut-off was 0.001 in both cases.

We tested whether there was residual population stratification due to mtDNA clustering within our working dataset of European/White individuals by plotting the top two principal components by mitochondrial lineage as reported by Erzurumluoglu et al. for Y chromosome haplogroups in ALSPAC [44].

All analyses were performed with the statistical package Stata 14 (StataCorp, College Station, TX, USA).

Statistical power was calculated with mitPower using binary variables, as this approach has not been developed for continuous or ordinal variables yet (http://bioinformatics.cesga.es/mitpower/) [45].

## 3. Results

Mitochondrial DNA haplogroups found in mothers and children of ALSPAC that were used in this study are shown in Table 1. For a more detailed haplogroup report see Table S3. The most frequent haplogroup was HV, representing almost 50% of the sample (49.3%), in agreement with the probable haplogroup composition of the UK estimated in a recent mtDNA study [46].

It is interesting to note, although not completely unexpected [47], that despite selecting individuals of European genomic ancestry and White ethnicity around 1% of them carried non-European mtDNA haplogroups (i.e., A, C, D, L, M, N) (Table S3), and were therefore excluded from the analysis. This can also be observed in individuals with an mtDNA quality score over 90% (Table S3). We uncovered no evidence of residual population stratification in the group of participants who carried a European mtDNA haplogroup (Figures S1 and S2).

No differences were identified in the distribution of mtDNA haplogroups by sex of the child (*p* = 0.15).

In the unadjusted analyses sample sizes ranged from 143 to 7629 in the mothers, and from 142 to 7373 in the children, whereas sample sizes for the adjusted analyses were between 100 and 4863, and between 137 and 6838, respectively.

Given the sample sizes available for the binary variables, the haplogroup frequencies and the number of haplogroups included in the analysis, we had 80% power to detect odds ratios (OR) of ~1.2 (ever smoked) to 1.7 (being a biological parent) at an α level of 0.05 if considering HV as the risk haplogroup. Under the same conditions, at the significance level corrected for multiple testing (*p* ≤ 0.001), those ORs become ~1.3 to 2.1. This calculation excludes the variable ‘having diabetes’ in children as there were only 26 subjects with the disease and mtDNA data in ALSPAC.

### 3.1. Association between Mitochondrial DNA Haplogroups and Breast Cancer Risk Factors in ALSPAC Mothers

In the unadjusted analysis 10 nominal associations of mtDNA haplogroups with breast cancer risk factors were found (*p* ≤ 0.05). Most of these associations involved body composition variables such as body mass index (BMI), weight, height and bone mineral density (Table S4). Seven associations were apparent after correction for age (or gestational age in the case of IGF and sex hormone measurements in pregnancy) and the top 10 principal components (Table 2). However, no strong evidence of association was present after applying a Bonferroni correction for multiple testing.

### 3.2. Association between Mitochondrial DNA Haplogroups and Breast Cancer Risk Factors in ALSPAC Children

Likewise, among the children no associations between mtDNA haplogroups and breast cancer risk factors were uncovered after multiple testing correction. All three associations showing a *p* ≤ 0.05 in unadjusted models were related to BMI and height (Table S5) and two of these were also detected after controlling for age, sex and the top 10 principal components (Table 3).

Similar results overall were obtained when using the more stringent quality score threshold of >90% (Table S6).

## 4. Discussion

In this study we have not found evidence that major mtDNA haplogroups underlie differences in breast cancer risk factor distribution. This finding in some way supports previous research showing that mtDNA lineages are not associated with breast cancer risk [23,48], although it is still possible that mtDNA variation directly affects cancer development without going through any of the risk factors investigated here. However, we did not observe any associations with cancer-specific traits available in the cohort, such as any cancer diagnosis in the mother or a breast cancer diagnosis in her biological mother. In addition, no association was evident with having had a mammogram either. Conversely, if mtDNA variation plays a role in breast cancer onset via any of the tested exposures it might represent a small increase in risk. Nevertheless, large scale case-control studies are needed to properly examine the association of mtDNA haplogroups with breast cancer.

We tried to reduce sources of bias such as small sample sizes and population stratification by using the ALSPAC cohort, where we had over 7700 individuals with mtDNA haplogroup data who were of European genomic ancestry or self-identified as being of White ethnicity. In addition, we ran regression models adjusted for principal components that reflect population structure in the South West region of the UK. Because ALSPAC has collected such a comprehensive set of phenotypes we were also able to examine most of the established and possible breast cancer risk factors.

Among the limitations of our study, the fact that a handful of traits examined had a low number of observations, in particular the hormone measures, decreased our confidence in these results. The minimum difference detectable with 80% power was an OR of 1.2 for the binary variables, as estimated using mitPower. In addition, some of the derived variables grouped all instances of a phenotype together, which may have prevented us from noticing an effect that depended on the frequency of such a phenotype.

Pre-Bonferroni correction, we detected associations of mtDNA haplogroups with body composition variables (mainly BMI, weight, height), which were seen in mothers as well as children and at various instances across the lifetime. A few earlier studies have shown an association of mtDNA haplogroups with obesity and obesity-related traits [17,49,50], while others reported no evidence of association [51,52]. In our analysis these associations have not survived multiple testing correction, so it is possible that they have arisen by chance or are the result of persisting population stratification (as different subsamples of mothers and children responded to questionnaires and were involved in the clinics), given that height and BMI are considerably structured across Europe [53]. On the other hand, we did not find any discernible stratification beyond what was accounted for by the use of genome-wide principal components, in agreement with a recent study that examined Y chromosome haplogroups in ALSPAC and showed that clustering by male lineage did not affect the association between autosomal single nucleotide polymorphisms (SNPs) and BMI [44].

Whilst we did not have a replication cohort to confirm our findings, the analysis was run in two groups of individuals, albeit related, each with a different set of phenotypes. Further investigation is needed to strengthen the results presented here; however, these could prove useful to generate hypotheses for future, more powerful studies.

## 5. Conclusions

Well-established and possible breast cancer risk factors were not found to be associated with mtDNA haplogroups in ALSPAC, a cohort of predominantly European ancestry. This study can serve as the basis for a further detailed analysis of the influence of mtDNA variation on nutritional, anthropometric and lifestyle exposures that underlie the susceptibility to breast cancer and other cancer types.

## Figures and Tables

**Table 1:** European mitochondrial DNA haplogroups in mothers and children of the Avon Longitudinal Study of Parents and Children (ALSPAC) used in the analysis of breast cancer risk factors (quality score > 80%).

mtDNA Haplogroups	N	%
HV	3835	49.3
U	1060	13.6
J	873	11.2
T	817	10.5
K	694	8.9
Other European	502	6.5
Total	7781	100.0

mtDNA: Mitochondrial DNA.

**Table 2:** Major European mitochondrial DNA (mtDNA) haplogroups and breast cancer risk factors in ALSPAC mothers. Regression models adjusted for age and the top 10 genomic principal components. (**A**) Continuous variables; (**B**) categorical variables. Reference is haplogroup HV.

A-Variable	Clade	Beta	95% CI	*p*-Value	*N*	Model *p*-Value
SHBG(nmol/L) in pregnancy ^1^	U	−40.28	(−136.56,56.01)	0.408	101	0.338
	J	−79.04	(−180.04,21.95)	0.123	101	
	T	40.96	(−48.11,130.02)	0.363	101	
	K	21.84	(−79.84,123.52)	0.670	101	
	Other European	−64.39	(−205.90,77.11)	0.368	101	
Testosterone (nmol/L) in pregnancy ^1^	U	−0.32	(−1.16,0.52)	0.444	100	0.228
	J	−0.10	(−0.98,0.77)	0.813	100	
	T	0.15	(−0.63,0.93)	0.705	100	
	K	0.98	(0.09,1.86)	0.031	100	
	Other European	−0.02	(−1.25,1.21)	0.972	100	
IGF-I (ng/mL) in pregnancy ^1^	U	4.21	(−19.23,27.66)	0.724	231	0.709
	J	2.53	(−21.74,26.81)	0.837	231	
	T	−2.67	(−25.40,20.05)	0.817	231	
	K	−14.90	(−42.78,12.99)	0.294	231	
	Other European	16.08	(−12.44,44.61)	0.268	231	
IGF-II (ng/mL) in pregnancy ^1^	U	34.45	(−51.06,119.97)	0.428	224	0.232
	J	−2.32	(−89.70,85.06)	0.958	224	
	T	−32.26	(−116.26,51.74)	0.450	224	
	K	105.67	(2.40,208.93)	0.045	224	
	Other European	61.39	(−41.29,164.06)	0.240	224	
IGFBP-3 (ng/mL) in pregnancy ^1^	U	−371.92	(−926.11,182.27)	0.187	231	0.023
	J	67.20	(−506.61,641.01)	0.818	231	
	T	−474.38	(−1011.50,62.74)	0.083	231	
	K	772.03	(112.93,1431.12)	0.022	231	
	Other European	381.81	(−292.51,1056.13)	0.266	231	
Mother’s natural mother’s age at birth (years)	U	0.08	(−0.44,0.60)	0.769	4164	0.985
	J	−0.04	(−0.61,0.52)	0.878	4164	
	T	0.14	(−0.42,0.70)	0.626	4164	
	K	0.18	(−0.43,0.78)	0.562	4164	
	Other European	0.11	(−0.60,0.83)	0.761	4164	
Age of mother at birth (years)	U	0.06	(−0.35,0.46)	0.791	4441	0.591
	J	0.32	(−0.13,0.76)	0.162	4441	
	T	−0.16	(−0.60,0.29)	0.493	4441	
	K	0.15	(−0.33,0.62)	0.549	4441	
	Other European	−0.17	(−0.73,0.39)	0.554	4441	
Age at menarche (years)	U	−0.02	(−0.16,0.12)	0.807	4073	0.043
	J	−0.17	(−0.32,−0.01)	0.032	4073	
	T	−0.19	(−0.35,−0.04)	0.013	4073	
	K	0.05	(−0.11,0.22)	0.535	4073	
	Other European	−0.11	(−0.30,0.09)	0.271	4073	
Age at menopause (years) (child ~20 years old)	U	0.95	(0.07,1.82)	0.034	877	0.329
	J	−0.09	(−1.02,0.84)	0.845	877	
	T	−0.16	(−1.20,0.89)	0.766	877	
	K	−0.15	(−1.12,0.83)	0.769	877	
	Other European	−0.16	(−1.37,1.06)	0.802	877	
Birth weight of mother (g)	U	−34.42	(−101.56,32.72)	0.315	2906	0.760
	J	−33.85	(−107.72,40.03)	0.369	2906	
	T	−6.93	(−78.36,64.50)	0.849	2906	
	K	−26.06	(−103.86,51.74)	0.511	2906	
	Other European	31.84	(−61.43,125.10)	0.503	2906	
BMI (~12 weeks gestation)	U	0.20	(−0.15,0.55)	0.260	4229	0.028
	J	0.60	(0.22,0.98)	0.002	4229	
	T	0.22	(−0.16,0.60)	0.259	4229	
	K	0.42	(0.02,0.83)	0.040	4229	
	Other European	0.02	(−0.47,0.50)	0.940	4229	
BMI (May–September 2010)	U	0.41	(−0.36,1.19)	0.299	1503	0.713
	J	0.45	(−0.38,1.27)	0.286	1503	
	T	−0.02	(−0.90,0.85)	0.956	1503	
	K	−0.28	(−1.16,0.60)	0.535	1503	
	Other European	0.18	(−0.83,1.19)	0.727	1503	
BMI (FOM1: 2009–2011)	U	0.27	(−0.35,0.90)	0.389	2565	0.175
	J	0.58	(−0.08,1.25)	0.086	2565	
	T	−0.44	(−1.13,0.25)	0.213	2565	
	K	0.32	(−0.40,1.05)	0.381	2565	
	Other European	0.54	(−0.32,1.39)	0.218	2565	
BMI (FOM2: 2011–2013)	U	0.68	(−0.09,1.45)	0.084	1649	0.438
	J	0.54	(−0.28,1.36)	0.195	1649	
	T	−0.13	(−1.02,0.75)	0.769	1649	
	K	0.06	(−0.84,0.95)	0.900	1649	
	Other European	0.40	(−0.61,1.42)	0.434	1649	
Pre-pregnancy weight (kg)	U	1.07	(0.07,2.07)	0.036	4272	0.035
	J	1.54	(0.46,2.62)	0.005	4272	
	T	0.95	(−0.14,2.04)	0.088	4272	
	K	1.01	(−0.15,2.17)	0.087	4272	
	Other European	0.50	(−0.88,1.88)	0.478	4272	
Weight (kg) (May–September 2010)	U	1.70	(−0.43,3.84)	0.118	1512	0.417
	J	0.77	(−1.51,3.05)	0.509	1512	
	T	−0.20	(−2.60,2.19)	0.869	1512	
	K	−1.16	(−3.59,1.27)	0.349	1512	
	Other European	1.18	(−1.62,3.98)	0.410	1512	
Weight (kg) (FOM1: 2009–2011)	U	1.10	(−0.63,2.83)	0.213	2565	0.288
	J	1.21	(−0.63,3.06)	0.198	2565	
	T	−0.50	(−2.41,1.41)	0.609	2565	
	K	0.61	(−1.39,2.61)	0.550	2565	
	Other European	2.18	(−0.18,4.53)	0.071	2565	
Weight (kg) (FOM2: 2011–2013)	U	2.53	(0.39,4.66)	0.020	1649	0.294
	J	1.20	(−1.05,3.45)	0.295	1649	
	T	0.79	(−1.65,3.22)	0.528	1649	
	K	0.52	(−1.95,3.00)	0.678	1649	
	Other European	1.52	(−1.28,4.32)	0.287	1649	
Height (cm) (~12 weeks gestation)	U	0.66	(0.05,1.27)	0.034	4434	0.134
	J	−0.26	(−0.91,0.40)	0.438	4434	
	T	0.36	(−0.30,1.02)	0.288	4434	
	K	−0.23	(−0.93,0.48)	0.529	4434	
	Other European	0.43	(−0.41,1.27)	0.315	4434	
Height (cm) (May–September 2010)	U	0.80	(−0.15,1.74)	0.098	1745	0.241
	J	−0.60	(−1.60,0.41)	0.244	1745	
	T	−0.09	(−1.14,0.97)	0.873	1745	
	K	−0.48	(−1.57,0.61)	0.389	1745	
	Other European	0.46	(−0.79,1.71)	0.467	1745	
Height (cm) (FOM1: 2009–2011)	U	0.48	(−0.25,1.21)	0.198	2565	0.060
	J	−0.40	(−1.18,0.38)	0.320	2565	
	T	0.91	(0.10,1.72)	0.028	2565	
	K	−0.29	(−1.14,0.56)	0.510	2565	
	Other European	0.68	(−0.32,1.68)	0.181	2565	
Sitting height (cm) (FOM1: 2009–2011)	U	0.36	(−0.03,0.75)	0.073	2545	0.057
	J	0.10	(−0.32,0.52)	0.637	2545	
	T	0.63	(0.20,1.06)	0.004	2545	
	K	0.05	(−0.40,0.50)	0.837	2545	
	Other European	0.35	(−0.18,0.88)	0.193	2545	
Leg length (cm) (FOM1: 2009–2011)	U	0.11	(−0.38,0.60)	0.670	2545	0.170
	J	−0.43	(−0.95,0.10)	0.111	2545	
	T	0.31	(−0.24,0.85)	0.270	2545	
	K	−0.34	(−0.90,0.23)	0.245	2545	
	Other European	0.36	(−0.30,1.03)	0.286	2545	
Height (cm) (FOM2: 2011–2013)	U	0.91	(0.00,1.81)	0.049	1649	0.041
	J	−0.38	(−1.33,0.58)	0.439	1649	
	T	1.36	(0.32,2.39)	0.010	1649	
	K	0.42	(−0.63,1.47)	0.434	1649	
	Other European	0.46	(−0.73,1.64)	0.449	1649	
Sitting height (cm) (FOM2: 2011–2013)	U	0.54	(0.09,1.00)	0.020	1648	0.016
	J	0.19	(−0.29,0.68)	0.432	1648	
	T	0.88	(0.35,1.40)	0.001	1648	
	K	0.26	(−0.27,0.80)	0.332	1648	
	Other European	0.35	(−0.25,0.96)	0.252	1648	
Leg length (cm) (FOM2: 2011–2013)	U	0.37	(−0.24,0.98)	0.239	1648	0.166
	J	−0.57	(−1.21,0.08)	0.084	1648	
	T	0.49	(−0.21,1.18)	0.173	1648	
	K	0.16	(−0.55,0.87)	0.660	1648	
	Other European	0.11	(−0.69,0.91)	0.788	1648	
Total body bone mineral density (FOM1: 2009–2011)	U	0.01	(−0.003,0.02)	0.156	2509	0.083
	J	−0.003	(−0.01,0.01)	0.549	2509	
	T	0.01	(0.003,0.03)	0.012	2509	
	K	−0.003	(−0.01,0.01)	0.601	2509	
	Other European	0.002	(−0.01,0.02)	0.744	2509	
Total body bone mineral density (FOM2: 2011–2013)	U	0.01	(−0.003,0.02)	0.146	1614	0.421
	J	−0.002	(−0.02,0.01)	0.820	1614	
	T	0.01	(−0.01,0.02)	0.378	1614	
	K	−0.01	(−0.02,0.01)	0.274	1614	
	Other European	0.001	(−0.02,0.02)	0.945	1614	
Daily fat intake (g) (~32 weeks gestation)	U	−0.36	(−1.13,0.41)	0.358	4386	0.230
	J	0.02	(−0.82,0.85)	0.971	4386	
	T	0.18	(−0.66,1.01)	0.678	4386	
	K	−0.08	(−0.98,0.81)	0.856	4386	
	Other European	−1.29	(−2.35,−0.23)	0.018	4386	
**B—Variable**	**Clade**	**OR**	**95% CI**	***p*-Value**	***N***	**Model *p*-Value**
parity (~18 weeks gestation)	U	1.01	(0.85,1.20)	0.907	4617	0.741
	J	1.07	(0.90,1.28)	0.447	4617	
	T	0.95	(0.79,1.15)	0.612	4617	
	K	1.01	(0.84,1.23)	0.885	4617	
	Other European	0.87	(0.68,1.09)	0.223	4617	
Number of pregnancies after study child (child ~21 months old)	U	0.95	(0.76,1.19)	0.639	4007	0.524
	J	0.82	(0.64,1.05)	0.119	4007	
	T	0.96	(0.75,1.22)	0.722	4007	
	K	0.80	(0.61,1.06)	0.123	4007	
	Other European	0.94	(0.69,1.27)	0.687	4007	
Alcohol consumption before this pregnancy	U	1.15	(0.97,1.36)	0.098	4645	0.082
	J	1.02	(0.85,1.22)	0.812	4645	
	T	0.94	(0.78,1.13)	0.495	4645	
	K	0.81	(0.66,0.98)	0.031	4645	
	Other European	0.95	(0.76,1.21)	0.695	4645	
Alcohol consumption (child ~8 months old)	U	0.94	(0.79,1.12)	0.500	4319	0.226
	J	0.88	(0.73,1.06)	0.169	4319	
	T	0.89	(0.73,1.07)	0.216	4319	
	K	0.79	(0.65,0.98)	0.032	4319	
	Other European	0.84	(0.66,1.07)	0.176	4319	
Quantity of alcohol mother drinks (child ~21 months old)	U	1.04	(0.87,1.23)	0.679	3995	0.170
	J	0.84	(0.70,1.03)	0.089	3995	
	T	0.95	(0.78,1.15)	0.584	3995	
	K	0.79	(0.64,0.98)	0.030	3995	
	Other European	0.96	(0.76,1.22)	0.767	3995	
Quantity of alcohol mother drinks (child ~33 months old)	U	0.98	(0.82,1.16)	0.796	3879	0.050
	J	0.86	(0.70,1.04)	0.124	3879	
	T	0.85	(0.70,1.03)	0.107	3879	
	K	0.73	(0.59,0.91)	0.006	3879	
	Other European	0.84	(0.65,1.07)	0.154	3879	
Quantity of alcohol mother drinks (child ~5 years old)	U	1.13	(0.93,1.38)	0.206	3445	0.058
	J	0.96	(0.79,1.17)	0.688	3445	
	T	0.91	(0.75,1.13)	0.399	3445	
	K	0.73	(0.59,0.92)	0.008	3445	
	Other European	1.00	(0.77,1.28)	0.972	3445	
Years taking contraceptive pill (child ~8 years old)	U	0.87	(0.69,1.09)	0.225	3056	0.170
	J	0.83	(0.64,1.05)	0.121	3056	
	T	1.12	(0.86,1.45)	0.425	3056	
	K	0.82	(0.63,1.07)	0.154	3056	
	Other European	1.16	(0.83,1.62)	0.389	3056	
Years taking contraceptive pill (child ~11 years old)	U	0.88	(0.70,1.09)	0.243	3352	0.289
	J	0.95	(0.74,1.21)	0.659	3352	
	T	1.00	(0.78,1.27)	0.974	3352	
	K	0.79	(0.61,1.03)	0.087	3352	
	Other European	1.22	(0.88,1.68)	0.233	3352	
Frequency of alcohol consumption (May–September 2010)	U	1.22	(0.93,1.60)	0.153	2036	0.425
	J	1.11	(0.84,1.48)	0.467	2036	
	T	0.90	(0.68,1.20)	0.483	2036	
	K	0.92	(0.68,1.25)	0.610	2036	
	Other European	1.22	(0.84,1.75)	0.292	2036	
Duration of breastfeeding last baby (~18 weeks gestation)	U	0.97	(0.78,1.22)	0.805	2508	0.229
	J	1.00	(0.79,1.28)	0.973	2508	
	T	1.22	(0.94,1.57)	0.127	2508	
	K	0.95	(0.73,1.23)	0.715	2508	
	Other European	1.39	(1.01,1.92)	0.044	2508	
Average time spent exercising in past year (from ~18 weeks gestation to 2010)	U	1.00	(0.85,1.19)	0.954	4545	0.392
	J	0.97	(0.80,1.16)	0.712	4545	
	T	1.00	(0.83,1.21)	0.996	4545	
	K	0.80	(0.66,0.98)	0.028	4545	
	Other European	0.94	(0.75,1.19)	0.633	4545	
Number of hours of activity per week (from ~18 weeks gestation to 2010)	U	0.93	(0.79,1.09)	0.378	4596	0.792
	J	0.93	(0.78,1.12)	0.452	4596	
	T	0.93	(0.78,1.13)	0.485	4596	
	K	0.96	(0.79,1.16)	0.648	4596	
	Other European	1.09	(0.86,1.38)	0.464	4596	
Number of hours of vigorous activity per week (from ~18 weeks gestation to 2010)	U	1.14	(0.95,1.36)	0.144	4670	0.684
	J	1.02	(0.84,1.25)	0.853	4670	
	T	1.08	(0.90,1.32)	0.396	4670	
	K	0.97	(0.78,1.20)	0.753	4670	
	Other European	0.98	(0.76,1.26)	0.860	4670	
Mother has reached menopause (child ~20 years old)	U	0.98	(0.69,1.39)	0.896	2074	0.442
	J	0.95	(0.65,1.38)	0.777	2074	
	T	0.73	(0.50,1.08)	0.112	2074	
	K	1.26	(0.85,1.88)	0.246	2074	
	Other European	1.07	(0.68,1.69)	0.761	2074	
Mother’s natural mother had breast cancer (~12 weeks gestation)	U	0.66	(0.38,1.14)	0.134	4509	0.679
	J	0.85	(0.50,1.44)	0.542	4509	
	T	0.83	(0.48,1.43)	0.494	4509	
	K	1.09	(0.65,1.83)	0.748	4509	
	Other European	1.04	(0.54,1.97)	0.915	4509	
Mother’s natural mother had breast cancer (child ~8 years old)	U	0.83	(0.52,1.33)	0.439	3122	0.496
	J	1.07	(0.67,1.71)	0.763	3122	
	T	0.61	(0.34,1.09)	0.094	3122	
	K	0.95	(0.56,1.60)	0.846	3122	
	Other European	0.68	(0.34,1.37)	0.282	3122	
Mother ever had a mammogram (FOM1: 2009–2011)	U	1.01	(0.66,1.55)	0.955	2577	0.408
	J	0.80	(0.50,1.28)	0.348	2577	
	T	0.73	(0.44,1.20)	0.218	2577	
	K	1.18	(0.72,1.91)	0.516	2577	
	Other European	0.63	(0.34,1.16)	0.137	2577	
Breastfed last baby (~18 weeks gestation)	U	1.04	(0.77,1.39)	0.814	2509	0.537
	J	1.11	(0.81,1.53)	0.519	2509	
	T	1.3	(0.93,1.83)	0.131	2509	
	K	1.05	(0.75,1.48)	0.767	2509	
	Other European	1.39	(0.90,2.14)	0.140	2509	
Mother ever had diabetes (from ~12 weeks gestation to 2010)	U	0.98	(0.58,1.67)	0.948	4772	0.428
	J	1.12	(0.65,1.93)	0.686	4772	
	T	1.67	(1.03,2.72)	0.039	4772	
	K	1.28	(0.73,2.23)	0.391	4772	
	Other European	1.03	(0.51,2.09)	0.934	4772	
Mother ever had any cancer (since child was ~6 years old to 2010)	U	0.60	(0.34,1.05)	0.071	4251	0.243
	J	0.67	(0.38,1.19)	0.171	4251	
	T	0.83	(0.49,1.41)	0.486	4251	
	K	1.04	(0.61,1.77)	0.882	4251	
	Other European	1.30	(0.74,2.29)	0.362	4251	
Mother ever had an hysterectomy and/or oophorectomy (since child was ~8 years old to 2010)	U	0.93	(0.60,1.43)	0.736	3728	0.955
	J	0.94	(0.60,1.49)	0.808	3728	
	T	1.15	(0.74,1.79)	0.526	3728	
	K	0.86	(0.51,1.45)	0.569	3728	
	Other European	0.96	(0.54,1.70)	0.884	3728	
Mother had ever done nightshift work (from ~18 weeks gestation to when child was ~10 years old)	U	1.05	(0.88,1.25)	0.590	4771	0.947
	J	0.96	(0.79,1.15)	0.636	4771	
	T	0.95	(0.79,1.16)	0.634	4771	
	K	0.95	(0.78,1.17)	0.648	4771	
	Other European	1.03	(0.81,1.31)	0.821	4771	
Mother ever took oral contraceptives (from ~12 weeks gestation to 2013)	U	1.17	(0.74,1.85)	0.491	4838	0.582
	J	1.07	(0.67,1.72)	0.776	4838	
	T	0.86	(0.55,1.33)	0.497	4838	
	K	0.77	(0.49,1.21)	0.252	4838	
	Other European	0.75	(0.45,1.27)	0.285	4838	
Mother ever used non-oral hormonal contraceptives (since child was ~21 months old to 2013)	U	1.04	(0.85,1.28)	0.687	4474	0.817
	J	0.93	(0.74,1.16)	0.528	4474	
	T	1.14	(0.92,1.42)	0.239	4474	
	K	1.02	(0.80,1.29)	0.901	4474	
	Other European	1.02	(0.77,1.35)	0.899	4474	
Mother ever had hormone replacement therapy (since child was ~9 years old to 2010)	U	1.06	(0.76,1.49)	0.724	3063	0.706
	J	0.99	(0.69,1.43)	0.971	3063	
	T	0.90	(0.61,1.33)	0.587	3063	
	K	1.32	(0.91,1.91)	0.138	3063	
	Other European	1.05	(0.67,1.67)	0.822	3063	
Mother ever taken aspirin (from ~18 weeks gestation to 2010)	U	1.02	(0.86,1.22)	0.807	4848	0.355
	J	1.08	(0.89,1.30)	0.445	4848	
	T	0.95	(0.78,1.15)	0.603	4848	
	K	0.81	(0.66,1.00)	0.051	4848	
	Other European	1.03	(0.81,1.30)	0.840	4848	
Mother ever smoked (from ~18 weeks gestation to 2010)	U	0.99	(0.83,1.17)	0.877	4863	0.739
	J	1.02	(0.85,1.23)	0.798	4863	
	T	0.93	(0.77,1.13)	0.458	4863	
	K	0.87	(0.71,1.07)	0.178	4863	
	Other European	0.92	(0.72,1.16)	0.478	4863	
Mother ever had an X-ray (from ~12 weeks gestation to when child was ~33 months old)	U	1.02	(0.84,1.24)	0.822	4631	0.640
	J	0.90	(0.73,1.11)	0.327	4631	
	T	0.97	(0.78,1.20)	0.773	4631	
	K	1.15	(0.93,1.44)	0.201	4631	
	Other European	0.96	(0.74,1.26)	0.777	4631	
Mother exercised at least once a week (from ~18 weeks gestation to 2010)	U	1.27	(0.95,1.69)	0.105	4723	0.071
	J	1.24	(0.91,1.69)	0.167	4723	
	T	1.01	(0.76,1.36)	0.924	4723	
	K	0.77	(0.58,1.02)	0.066	4723	
	Other European	1.19	(0.81,1.76)	0.374	4723	

^1^ Adjusted for gestational age and 10 principal components. FOM: Focus on Mothers clinic; SHBG: Sex hormone binding globulin; IGF:Insulin-like growth factor; IGFBP: Insulin-like growth factor binding protein; BMI: Body mass index; ALSPAC: Avon Longitudinal Study of Parents and Children.

**Table 3:** Major European mtDNA haplogroups and breast cancer risk factors in ALSPAC children. Regression models adjusted for age, sex and the top 10 genomic principal components. (**A**) Continuous variables; (**B**) categorical variables. Reference is haplogroup HV.

A—Variable	Clade	Beta	95% CI	*p*-Value	*N*	Model *p*-Value
SHBG (nmol/L) (~8 years old)	U	−3.13	(−10.91,4.65)	0.429	635	0.435
	J	1.05	(−6.84,8.94)	0.794	635	
	T	−8.19	(−16.27,−0.11)	0.047	635	
	K	−0.32	(−8.93,8.28)	0.941	635	
	Other European	−2.52	(−12.58,7.53)	0.623	635	
SHBG, nmol/L) (~15 years old) ^1^	U	−0.71	(−5.93,4.52)	0.790	1281	0.812
	J	−3.64	(−9.15,1.87)	0.195	1281	
	T	−1.67	(−7.14,3.8)	0.550	1281	
	K	0.85	(−5.29,6.98)	0.786	1281	
	Other European	−1.95	(−8.86,4.96)	0.580	1281	
Testosterone (nmol/L) (~15 years old) ^1^	U	0.01	(−0.06,0.08)	0.775	1339	0.478
	J	−0.05	(−0.13,0.02)	0.172	1339	
	T	−0.004	(−0.08,0.07)	0.911	1339	
	K	0.05	(−0.04,0.14)	0.242	1339	
	Other European	−0.04	(−0.14,0.06)	0.463	1339	
Androstenedione (ng/dL) (~8 years old)	U	0.97	(−6.49,8.42)	0.799	583	0.948
	J	−0.64	(−8.18,6.9)	0.867	583	
	T	2.75	(−5.10,10.61)	0.492	583	
	K	−1.13	(−9.24,6.97)	0.783	583	
	Other European	−2.80	(−12.70,7.09)	0.578	583	
DHEAS(ug/dL) (~8 years old) ^2^	U	−0.02	(−7.00,6.95)	0.995	585	0.269
	J	−1.83	(−8.91,5.26)	0.613	585	
	T	6.29	(−1.04,13.63)	0.093	585	
	K	−3.89	(−11.51,3.73)	0.317	585	
	Other European	−5.05	(−14.35,4.25)	0.286	585	
IGF-I (ng/mL) (cord blood) ^3^	U	6.38	(−4.09,16.85)	0.232	442	0.125
	J	11.50	(−0.87,23.87)	0.068	442	
	T	−3.09	(−14.72,8.54)	0.602	442	
	K	6.36	(−6.65,19.37)	0.337	442	
	Other European	14.71	(0.55,28.87)	0.042	442	
IGF-I (~7 years old)	U	−6.19	(−22.93,10.55)	0.468	340	0.347
	J	8.42	(−8.53,25.37)	0.329	340	
	T	−13.79	(−31.15,3.57)	0.119	340	
	K	3.32	(−16.52,23.16)	0.742	340	
	Other European	−10.36	(−32.65,11.92)	0.361	340	
IGF-I (ng/mL) (~8 years old)	U	6.41	(−13.53,26.34)	0.528	317	0.499
	J	−19.44	(−42.05,3.18)	0.092	317	
	T	−2.37	(−22.9,18.16)	0.821	317	
	K	−5.54	(−27.13,16.05)	0.614	317	
	Other European	2.74	(−25.34,30.81)	0.848	317	
IGF-II (ng/mL) (cord blood) ^3^	U	−9.90	(−32.12,12.32)	0.382	443	0.032
	J	−24.76	(−51.01,1.49)	0.064	443	
	T	23.21	(−1.46,47.89)	0.065	443	
	K	−24.67	(−52.29,2.95)	0.080	443	
	Other European	−3.16	(−33.22,26.89)	0.836	443	
IGF-II (ng/mL) (~7 years old)	U	−33.08	(−95.43,29.26)	0.296	149	0.647
	J	−13.87	(−69.00,41.26)	0.620	149	
	T	−53.36	(−120,12.9519)	0.114	149	
	K	−8.39	(−77.90,61.13)	0.812	149	
	Other European	4.21	(−74.08,82.51)	0.915	149	
IGFBP-3 (ng/mL) (cord blood) ^3^	U	−60.05	(−346.54,226.44)	0.679	137	0.856
	J	−65.89	(−413.24,281.45)	0.708	137	
	T	132.24	(−118.57,383.05)	0.299	137	
	K	−37.07	(−352.6,278.46)	0.816	137	
	Other European	−26.71	(−376.79,323.37)	0.880	137	
IGFBP-3 (ng/mL) (~7 years old)	U	−77.05	(−440,29.44)	0.679	340	0.439
	J	76.89	(−290.00,447.99)	0.684	340	
	T	−320.00	(−700,63.7442)	0.103	340	
	K	−180.00	(−620.00,251.58)	0.408	340	
	Other European	−320.00	(−810.00,169.81)	0.201	340	
IGFBP-3 (ng/mL) (~8 years old)	U	22.75	(−572.91,618.40)	0.940	317	0.674
	J	77.72	(−598.04,753.47)	0.821	317	
	T	150.74	(−462.65,764.13)	0.629	317	
	K	404.59	(−240.45,1049.63)	0.218	317	
	Other European	−412.02	(−1253.45,429.42)	0.336	317	
Age at menarche (months) ^1^	U	1.30	(0.29,2.89)	0.108	2728	0.254
	J	−0.85	(−2.56,0.86)	0.332	2728	
	T	0.08	(−1.64,1.79)	0.931	2728	
	K	−1.30	(−3.30,0.69)	0.201	2728	
	Other European	−0.56	(−2.75,1.63)	0.616	2728	
BMI (kg/m^2^) (~12 months old)	U	0.15	(−0.14,0.44)	0.313	773	0.014
	J	−0.13	(−0.48,0.22)	0.478	773	
	T	0.25	(−0.05,0.54)	0.099	773	
	K	−0.34	(−0.67,0.00)	0.047	773	
	Other European	0.40	(0.02,0.79)	0.041	773	
BMI (kg/m^2^) (~25 months old)	U	0.13	(−0.19,0.44)	0.435	675	0.507
	J	−0.16	(−0.54,0.23)	0.427	675	
	T	0.21	(−0.12,0.53)	0.213	675	
	K	−0.06	(−0.44,0.31)	0.746	675	
	Other European	0.22	(−0.21,0.65)	0.310	675	
BMI (kg/m^2^) (~37 months old)	U	0.12	(−0.18,0.43)	0.429	691	0.143
	J	−0.21	(−0.58,0.15)	0.254	691	
	T	0.13	(−0.18,0.45)	0.410	691	
	K	0.12	(−0.24,0.48)	0.518	691	
	Other European	0.48	(0.06,0.89)	0.024	691	
BMI (kg/m^2^) (~61 months old)	U	0.13	(−0.23,0.48)	0.488	664	0.103
	J	−0.08	(−0.50,0.35)	0.725	664	
	T	0.28	(−0.07,0.64)	0.118	664	
	K	−0.05	(−0.45,0.36)	0.827	664	
	Other European	0.61	(0.14,1.08)	0.012	664	
BMI (kg/m^2^) (~7 years old)	U	−0.04	(−0.21,0.13)	0.647	5345	0.898
	J	−0.03	(−0.21,0.15)	0.721	5345	
	T	0.01	(−0.18,0.19)	0.945	5345	
	K	0.09	(−0.11,0.29)	0.378	5345	
	Other European	0.06	(−0.17,0.28)	0.611	5345	
BMI (kg/m^2^) (~9 years old)	U	0.01	(−0.23,0.25)	0.943	5106	0.981
	J	0.03	(−0.22,0.28)	0.822	5106	
	T	−0.10	(−0.36,0.16)	0.456	5106	
	K	−0.01	(−0.29,0.28)	0.970	5106	
	Other European	0.01	(−0.31,0.32)	0.968	5106	
BMI (kg/m^2^) (~11 years old)	U	−0.09	(−0.38,0.20)	0.551	4764	0.768
	J	0.01	(−0.30,0.33)	0.927	4764	
	T	0.01	(−0.31,0.33)	0.940	4764	
	K	−0.03	(−0.38,0.32)	0.864	4764	
	Other European	0.27	(−0.12,0.66)	0.179	4764	
BMI (kg/m^2^) (~13 years old)	U	0.004	(−0.30,0.31)	0.981	4516	0.787
	J	−0.05	(−0.39,0.28)	0.755	4516	
	T	−0.02	(−0.36,0.32)	0.905	4516	
	K	−0.24	(−0.61,0.13)	0.209	4516	
	Other European	0.15	(−0.27,0.56)	0.483	4516	
BMI (kg/m^2^) (~15 years old)	U	−0.08	(−0.43,0.27)	0.656	3694	0.219
	J	0.13	(−0.24,0.49)	0.499	3694	
	T	−0.18	(−0.56,0.20)	0.354	3694	
	K	−0.04	(−0.44,0.36)	0.842	3694	
	Other European	0.49	(0.04,0.95)	0.033	3694	
BMI (kg/m^2^) (~17 years old)	U	−0.15	(−0.56,0.27)	0.490	3327	0.812
	J	0.17	(−0.27,0.62)	0.445	3327	
	T	−0.02	(−0.47,0.43)	0.931	3327	
	K	−0.10	(−0.58,0.39)	0.700	3327	
	Other European	0.24	(−0.33,0.80)	0.417	3327	
BMI (kg/m^2^) (~11 years old, puberty questionnaire)	U	−0.04	(−0.40,0.31)	0.813	2874	0.583
	J	0.03	(−0.34,0.39)	0.886	2874	
	T	0.03	(−0.35,0.41)	0.878	2874	
	K	0.36	(−0.07,0.79)	0.099	2874	
	Other European	−0.17	(−0.63,0.30)	0.479	2874	
BMI (kg/m^2^) (~13 years old, puberty questionnaire)	U	−0.11	(−0.49,0.26)	0.554	2889	0.604
	J	−0.22	(−0.62,0.19)	0.292	2889	
	T	−0.19	(−0.60,0.22)	0.355	2889	
	K	−0.36	(−0.81,0.10)	0.126	2889	
	Other European	0.05	(−0.46,0.55)	0.861	2889	
BMI (kg/m^2^) (~15 years old, puberty questionnaire)	U	−0.06	(−0.44,0.33)	0.768	2505	0.456
	J	0.25	(−0.16,0.66)	0.237	2505	
	T	−0.05	(−0.47,0.37)	0.825	2505	
	K	−0.15	(−0.62,0.32)	0.539	2505	
	Other European	0.40	(−0.12,0.92)	0.135	2505	
BMI (kg/m^2^) (~17 years old, puberty questionnaire)	U	−0.24	(−0.66,0.19)	0.281	2374	0.123
	J	0.38	(−0.06,0.83)	0.093	2374	
	T	0.21	(−0.25,0.67)	0.373	2374	
	K	−0.03	(−0.54,0.48)	0.904	2374	
	Other European	0.52	(−0.05,1.09)	0.072	2374	
Birth weight (g)	U	−7.46	(−39.89,24.96)	0.652	6838	0.584
	J	−5.43	(−40.22,29.36)	0.759	6838	
	T	−25.25	(−61.23,10.74)	0.169	6838	
	K	−12.34	(−50.81,26.12)	0.529	6838	
	Other European	−33.86	(−77.51,9.79)	0.128	6838	
Weight (kg) (~12 months old)	U	0.10	(−0.13,0.34)	0.396	775	0.092
	J	−0.05	(−0.34,0.23)	0.718	775	
	T	0.22	(−0.03,0.46)	0.079	775	
	K	−0.24	(−0.51,0.03)	0.081	775	
	Other European	0.16	(−0.15,0.48)	0.309	775	
Weight (kg) (~25 months old)	U	0.15	(−0.17,0.48)	0.358	718	0.301
	J	0.0002	(−0.39,0.39)	0.999	718	
	T	0.31	(−0.01,0.64)	0.061	718	
	K	−0.19	(−0.57,0.19)	0.317	718	
	Other European	0.02	(−0.42,0.45)	0.938	718	
Weight (kg) (~37 months old)	U	0.20	(−0.20,0.60)	0.330	699	0.745
	J	−0.09	(−0.58,0.39)	0.704	699	
	T	0.22	(−0.20,0.64)	0.301	699	
	K	−0.01	(−0.48,0.46)	0.966	699	
	Other European	0.24	(−0.31,0.78)	0.395	699	
Weight (kg) (~61 months old)	U	0.40	(−0.25,1.05)	0.229	667	0.494
	J	0.18	(−0.59,0.95)	0.648	667	
	T	0.53	(−0.13,1.18)	0.113	667	
	K	−0.18	(−0.93,0.56)	0.626	667	
	Other European	0.31	(−0.56,1.18)	0.484	667	
Weight (kg) (~7 years old)	U	−0.11	(−0.48,0.26)	0.573	5346	0.974
	J	0.03	(−0.37,0.42)	0.896	5346	
	T	−0.02	(−0.42,0.39)	0.937	5346	
	K	0.13	(−0.32,0.57)	0.573	5346	
	Other European	−0.05	(−0.54,0.45)	0.846	5346	
Weight (kg) (~9 years old)	U	0.01	(−0.60,0.62)	0.981	5149	0.877
	J	0.28	(−0.37,0.92)	0.402	5149	
	T	0.03	(−0.63,0.69)	0.937	5149	
	K	0.04	(−0.68,0.76)	0.909	5149	
	Other European	−0.37	(−1.18,0.44)	0.368	5149	
Weight (kg, DEXA) (~9 years old)	U	0.04	(−0.57,0.65)	0.892	4910	0.802
	J	0.36	(−0.28,1.01)	0.273	4910	
	T	0.03	(−0.63,0.69)	0.931	4910	
	K	−0.03	(−0.76,0.69)	0.931	4910	
	Other European	−0.36	(−1.18,0.45)	0.386	4910	
Weight (kg) (~11 years old)	U	−0.17	(−1.02,0.67)	0.690	4770	0.972
	J	0.21	(−0.70,1.13)	0.646	4770	
	T	0.15	(−0.78,1.09)	0.748	4770	
	K	0.14	(−0.88,1.16)	0.787	4770	
	Other European	0.31	(−0.83,1.45)	0.595	4770	
Weight (kg) (~13 years old)	U	0.18	(−0.77,1.13)	0.715	4516	0.931
	J	0.30	(−0.73,1.33)	0.568	4516	
	T	0.42	(−0.62,1.46)	0.430	4516	
	K	−0.28	(−1.43,0.87)	0.631	4516	
	Other European	0.08	(−1.20,1.36)	0.905	4516	
Weight (kg) (~15 years old)	U	−0.07	(−1.20,1.07)	0.910	3694	0.242
	J	0.91	(−0.29,2.12)	0.136	3694	
	T	−0.41	(−1.66,0.83)	0.513	3694	
	K	0.04	(−1.29,1.36)	0.957	3694	
	Other European	1.45	(−0.05,2.94)	0.057	3694	
Weight (kg) (~17 years old)	U	−0.21	(−1.52,1.11)	0.756	3329	0.902
	J	0.59	(−0.82,2.00)	0.411	3329	
	T	0.05	(−1.38,1.48)	0.945	3329	
	K	−0.05	(−1.58,1.49)	0.953	3329	
	Other European	0.77	(−1.03,2.56)	0.402	3329	
Weight (kg) (~11 years old, puberty questionnaire)	U	0.18	(−0.71,1.07)	0.693	3153	0.757
	J	0.44	(−0.49,1.36)	0.356	3153	
	T	0.18	(−0.77,1.13)	0.714	3153	
	K	0.57	(−0.51,1.65)	0.300	3153	
	Other European	−0.42	(−1.61,0.76)	0.484	3153	
Weight (kg) (~13 years old, puberty questionnaire)	U	0.51	(−0.62,1.64)	0.373	3053	0.959
	J	0.08	(−1.11,1.28)	0.891	3053	
	T	0.30	(−0.92,1.52)	0.629	3053	
	K	0.00	(−1.37,1.36)	0.998	3053	
	Other European	0.36	(−1.17,1.89)	0.644	3053	
Weight (kg) (~15 years old, puberty questionnaire)	U	−0.29	(−1.52,0.95)	0.649	2659	0.567
	J	0.77	(−0.55,2.09)	0.255	2659	
	T	0.19	(−1.17,1.55)	0.787	2659	
	K	0.41	(−1.10,1.92)	0.596	2659	
	Other European	1.25	(−0.43,2.93)	0.145	2659	
Weight (kg) (~17 years old, puberty questionnaire)	U	−0.27	(−1.64,1.10)	0.699	2476	0.092
	J	1.82	(0.39,3.24)	0.012	2476	
	T	0.45	(−1.03,1.93)	0.549	2476	
	K	0.20	(−1.45,1.85)	0.811	2476	
	Other European	1.60	(−0.20,3.40)	0.081	2476	
Height (cm) (~25 months old)	U	−0.05	(−0.75,0.64)	0.881	675	0.130
	J	0.22	(−0.62,1.07)	0.604	675	
	T	0.32	(−0.39,1.03)	0.384	675	
	K	−0.64	(−1.47,0.18)	0.125	675	
	Other European	−0.99	(−1.93,−0.05)	0.040	675	
Height (cm) (~37 months old)	U	0.23	(−0.57,1.03)	0.579	691	0.547
	J	0.28	(−0.67,1.24)	0.561	691	
	T	0.15	(−0.68,0.98)	0.724	691	
	K	−0.51	(−1.45,0.43)	0.286	691	
	Other European	−0.67	(−1.76,0.41)	0.222	691	
Height (cm) (~61 months old)	U	0.67	(−0.37,1.71)	0.206	665	0.134
	J	0.79	(−0.44,2.02)	0.207	665	
	T	0.44	(−0.6,1.48)	0.407	665	
	K	−0.46	(−1.64,0.73)	0.447	665	
	Other European	−1.18	(−2.56,0.2)	0.093	665	
Sitting height (kg) (~61 months old)	U	0.33	(−0.25,0.9)	0.262	667	0.722
	J	0.21	(−0.47,0.89)	0.545	667	
	T	0.15	(−0.43,0.72)	0.617	667	
	K	−0.13	(−0.79,0.52)	0.694	667	
	Other European	−0.28	(−1.04,0.48)	0.472	667	
Height (cm) (~7 years old)	U	−0.07	(−0.50,0.36)	0.752	5350	0.885
	J	0.22	(−0.25,0.68)	0.365	5350	
	T	−0.03	(−0.51,0.44)	0.890	5350	
	K	−0.03	(−0.55,0.49)	0.915	5350	
	Other European	−0.21	(−0.79,0.37)	0.484	5350	
Sitting height (cm) (~7 years old)	U	−0.02	(−0.24,0.21)	0.878	5351	0.949
	J	0.03	(−0.21,0.27)	0.821	5351	
	T	0.01	(−0.24,0.25)	0.966	5351	
	K	−0.02	(−0.29,0.25)	0.884	5351	
	Other European	−0.15	(−0.45,0.15)	0.324	5351	
Height (cm) (~9 years old)	U	0.07	(−0.44,0.59)	0.778	5108	0.213
	J	0.49	(−0.06,1.04)	0.081	5108	
	T	0.33	(−0.23,0.89)	0.247	5108	
	K	0.19	(−0.43,0.80)	0.551	5108	
	Other European	−0.49	(−1.18,0.20)	0.165	5108	
Sitting height (cm) (~9 years old)	U	0.11	(−0.15,0.37)	0.397	5145	0.178
	J	0.28	(0.01,0.55)	0.042	5145	
	T	0.17	(−0.10,0.45)	0.221	5145	
	K	0.06	(−0.24,0.37)	0.680	5145	
	Other European	−0.20	(−0.54,0.14)	0.252	5145	
Height (cm) (~11 years old)	U	−0.02	(−0.63,0.59)	0.946	4765	0.614
	J	0.32	(−0.34,0.98)	0.342	4765	
	T	0.22	(−0.45,0.90)	0.517	4765	
	K	0.36	(−0.38,1.10)	0.342	4765	
	Other European	−0.43	(−1.26,0.39)	0.304	4765	
Sitting height (cm) (~11 years old)	U	−0.08	(−0.40,0.23)	0.604	4769	0.784
	J	0.10	(−0.24,0.44)	0.566	4769	
	T	0.10	(−0.25,0.45)	0.558	4769	
	K	0.11	(−0.28,0.49)	0.587	4769	
	Other European	−0.20	(−0.63,0.23)	0.362	4769	
Height (cm) (~13 years old)	U	0.18	(−0.49,0.84)	0.601	4559	0.162
	J	0.67	(−0.05,1.39)	0.069	4559	
	T	0.67	(−0.06,1.39)	0.072	4559	
	K	0.46	(−0.34,1.26)	0.262	4559	
	Other European	−0.39	(−1.28,0.51)	0.394	4559	
Sitting height (cm) (~13 years old)	U	0.07	(−0.29,0.43)	0.694	4531	0.106
	J	0.34	(−0.05,0.73)	0.087	4531	
	T	0.35	(−0.04,0.74)	0.080	4531	
	K	0.29	(−0.15,0.72)	0.195	4531	
	Other European	−0.31	(−0.79,0.17)	0.206	4531	
Height (cm) (~15 years old)	U	0.21	(−0.47,0.89)	0.551	3702	0.514
	J	0.75	(0.02,1.47)	0.043	3702	
	T	0.16	(−0.58,0.90)	0.667	3702	
	K	0.22	(−0.57,1.01)	0.589	3702	
	Other European	0.03	(−0.86,0.93)	0.940	3702	
Sitting height (cm) (~15 years old)	U	−0.07	(−0.47,0.33)	0.739	3139	0.188
	J	0.52	(0.09,0.94)	0.017	3139	
	T	0.11	(−0.33,0.54)	0.630	3139	
	K	0.15	(−0.32,0.62)	0.537	3139	
	Other European	−0.17	(−0.71,0.37)	0.534	3139	
Height (cm) (~17 years old)	U	0.23	(−0.44,0.91)	0.496	3330	0.975
	J	0.14	(−0.58,0.86)	0.701	3330	
	T	0.15	(−0.59,0.88)	0.693	3330	
	K	0.27	(−0.52,1.05)	0.507	3330	
	Other European	0.06	(−0.86,0.98)	0.904	3330	
Height (cm) (~11 years old, puberty questionnaire)	U	0.32	(−0.45,1.10)	0.415	3167	0.656
	J	0.45	(−0.35,1.26)	0.271	3167	
	T	0.56	(−0.28,1.39)	0.191	3167	
	K	0.20	(−0.74,1.14)	0.677	3167	
	Other European	−0.21	(−1.23,0.80)	0.680	3167	
Height (cm) (~13 years old, puberty questionnaire)	U	1.09	(0.19,1.98)	0.017	3277	0.020
	J	1.19	(0.23,2.14)	0.015	3277	
	T	1.22	(0.26,2.19)	0.013	3277	
	K	0.71	(−0.37,1.79)	0.200	3277	
	Other European	0.14	(−1.06,1.33)	0.821	3277	
Height (cm) (~15 years old, puberty questionnaire)	U	0.27	(−0.63,1.16)	0.559	2797	0.502
	J	0.34	(−0.62,1.30)	0.482	2797	
	T	0.33	(−0.66,1.31)	0.517	2797	
	K	1.10	(−0.02,2.23)	0.054	2797	
	Other European	−0.15	(−1.37,1.07)	0.809	2797	
Height (cm) (~17 years old, puberty questionnaire)	U	0.11	(−0.74,0.95)	0.802	2567	0.846
	J	0.61	(−0.28,1.51)	0.179	2567	
	T	−0.03	(−0.94,0.88)	0.944	2567	
	K	0.18	(−0.84,1.19)	0.729	2567	
	Other European	−0.06	(−1.19,1.07)	0.918	2567	
BMD (g/cm^2^) (~9 years old)	U	−0.002	(−0.006,0.003)	0.482	4910	0.691
	J	−0.0005	(−0.005,0.004)	0.845	4910	
	T	0.003	(−0.002,0.008)	0.182	4910	
	K	0.001	(−0.004,0.006)	0.622	4910	
	Other European	0.0002	(−0.006,0.006)	0.939	4910	
BMD (g/cm^2^) (~11 years old)	U	−0.002	(−0.007,0.003)	0.461	4699	0.498
	J	0.001	(−0.005,0.007)	0.740	4699	
	T	0.004	(−0.002,0.010)	0.159	4699	
	K	0.004	(−0.003,0.010)	0.252	4699	
	Other European	0.002	(−0.006,0.009)	0.656	4699	
BMD (g/cm^2^ (~13 years old)	U	−0.003	(−0.010,0.005)	0.489	3903	0.635
	J	0.004	(−0.004,0.012)	0.333	3903	
	T	0.004	(−0.004,0.013)	0.298	3903	
	K	0.004	(−0.005,0.013)	0.389	3903	
	Other European	0.001	(−0.009,0.011)	0.807	3903	
BMD (g/cm^2^) (~15 years old)	U	−0.006	(−0.015,0.002)	0.147	3550	0.206
	J	0.004	(−0.005,0.012)	0.432	3550	
	T	0.004	(−0.005,0.013)	0.362	3550	
	K	0.005	(−0.005,0.015)	0.311	3550	
	Other European	0.008	(−0.004,0.019)	0.183	3550	
BMD (g/cm^2^) (~17 years old)	U	−0.001	(−0.001,0.008)	0.884	3219	0.866
	J	0.001	(−0.009,0.010)	0.840	3219	
	T	0.006	(−0.004,0.016)	0.251	3219	
	K	0.001	(−0.010,0.011)	0.863	3219	
	Other European	0.005	(−0.008,0.017)	0.459	3219	
**B—Variable**	**Clade**	**OR**	**95% CI**	***p*-Value**	***N***	**model *p*-Value**
YYP was breastfed as a baby	U	1.08	(0.94,1.26)	0.276	6357	0.630
	J	1.13	(0.96,1.31)	0.139	6357	
	T	1.02	(0.87,1.20)	0.783	6357	
	K	1.01	(0.85,1.20)	0.895	6357	
	Other European	0.96	(0.79,1.17)	0.672	6357	
Frequency YP has a drink containing alcohol (~17 years old, questionnaire)	U	0.99	(0.81,1.21)	0.897	3126	0.893
	J	1.08	(0.88,1.35)	0.432	3126	
	T	1.04	(0.83,1.30)	0.754	3126	
	K	1.08	(0.85,1.38)	0.524	3126	
	Other European	1.14	(0.87,1.48)	0.359	3126	
Frequency YP has a drink containing alcohol (~17 years old, clinic visit)	U	1.12	(0.90,1.39)	0.314	2655	0.629
	J	1.21	(0.96,1.52)	0.107	2655	
	T	1.04	(0.82,1.32)	0.761	2655	
	K	1.00	(0.78,1.28)	0.996	2655	
	Other European	1.13	(0.83,1.52)	0.454	2655	
Frequency respondent has a drink containing alcohol (~19 years old)	U	0.94	(0.74,1.20)	0.615	2136	0.862
	J	1.07	(0.84,1.39)	0.572	2136	
	T	0.96	(0.73,1.26)	0.770	2136	
	K	1.15	(0.86,1.55)	0.337	2136	
	Other European	1.04	(0.76,1.45)	0.806	2136	
Over the past year frequency YP had a drink containing alcohol (~21 years old)	U	1.06	(0.86,1.30)	0.578	2701	0.568
	J	1.11	(0.89,1.38)	0.385	2701	
	T	1.11	(0.87,1.40)	0.430	2701	
	K	0.91	(0.70,1.17)	0.477	2701	
	Other European	1.23	(0.91,1.67)	0.166	2701	
Over the past year frequency YP had a drink containing alcohol (~23 years old)	U	0.87	(0.70,1.08)	0.202	2499	0.710
	J	1.03	(0.82,1.30)	0.804	2499	
	T	0.98	(0.77,1.25)	0.853	2499	
	K	0.86	(0.66,1.12)	0.257	2499	
	other European	0.99	(0.72,1.35)	0.935	2499	
Frequency of physical activity during the past month (8–12 years old)	U	0.94	(0.80,1.12)	0.507	5556	0.679
	J	0.97	(0.81,1.16)	0.752	5556	
	T	1.06	(0.89,1.28)	0.500	5556	
	K	1.12	(0.91,1.36)	0.262	5556	
	Other European	1.08	(0.87,1.35)	0.474	5556	
Frequency of physical activity during the past month (13–18 years old)	U	0.94	(0.80,1.11)	0.460	5573	0.851
	J	0.92	(0.78,1.08)	0.329	5573	
	T	0.94	(0.79,1.12)	0.505	5573	
	K	1.04	(0.86,1.26)	0.669	5573	
	Other European	0.99	(0.80,1.22)	0.924	5573	
Frequency of physical activity during the past year (13–18 years old)	U	1.02	(0.86,1.21)	0.854	4540	0.906
	J	1.03	(0.86,1.23)	0.772	4540	
	T	1.01	(0.84,1.22)	0.904	4540	
	K	1.14	(0.92,1.39)	0.228	4540	
	Other European	1.05	(0.84,1.32)	0.646	4540	
YP was breastfed as a baby	U	0.91	(0.75,1.10)	0.324	6383	0.820
	J	0.92	(0.75,1.12)	0.393	6383	
	T	0.93	(0.75,1.15)	0.498	6383	
	K	1.03	(0.81,1.30)	0.826	6383	
	Other European	1.05	(0.80,1.36)	0.740	6383	
YP is a biological parent (21–23 years old)	U	0.55	(0.31,0.98)	0.043	3563	0.205
	J	0.92	(0.55,1.54)	0.760	3563	
	T	0.94	(0.56,1.59)	0.830	3563	
	K	0.60	(0.30,1.21)	0.155	3563	
	Other European	1.31	(0.73,2.35)	0.374	3563	
YP ever took oral contraceptives (from 8 to 22 years old)	U	1.05	(0.85,1.29)	0.665	3483	0.512
	J	1.13	(0.91,1.41)	0.273	3483	
	T	1.02	(0.81,1.27)	0.886	3483	
	K	1.16	(0.90,1.49)	0.254	3483	
	Other European	0.85	(0.64,1.13)	0.252	3483	
YP ever used non-oral hormonal contraceptives (from 17 to 22 years old)	U	1.32	(0.95,1.82)	0.096	2709	0.529
	J	1.11	(0.78,1.58)	0.572	2709	
	T	0.94	(0.64,1.38)	0.749	2709	
	K	1.09	(0.73,1.64)	0.659	2709	
	Other European	0.85	(0.51,1.42)	0.529	2709	
Teenager has drunk alcohol (~13 years old)	U	1.09	(0.88,1.35)	0.436	3117	0.817
	J	1.09	(0.86,1.37)	0.484	3117	
	T	0.96	(0.75,1.22)	0.729	3117	
	K	1.01	(0.78,1.31)	0.945	3117	
	Other European	0.88	(0.65,1.19)	0.408	3117	
YP has ever drunk alcohol (~17 years old)	U	1.00	(0.64,1.57)	0.998	3316	0.962
	J	1.17	(0.70,1.95)	0.549	3316	
	T	1.21	(0.71,2.07)	0.479	3316	
	K	1.05	(0.60,1.81)	0.871	3316	
	Other European	0.92	(0.52,1.62)	0.762	3316	
YP ever smoked (from 14 to 23 years old)	U	1.16	(0.98,1.38)	0.088	5615	0.262
	J	1.06	(0.88,1.27)	0.564	5615	
	T	1.06	(0.88,1.28)	0.524	5615	
	K	1.14	(0.93,1.40)	0.210	5615	
	Other European	0.88	(0.70,1.10)	0.247	5615	
YP ever had diabetes (from 11 to 23 years old)	U	3.50	(1.22,10.06)	0.020	3895	0.257
	J	3.05	(0.96,9.70)	0.058	3895	
	T	1.92	(0.49,7.48)	0.345	3895	
	K	1.50	(0.31,7.28)	0.614	3895	
	Other European	2.07	(0.42,10.08)	0.369	3895	
YP ever had an X-ray (from 6 months to 15 years old)	U	1.04	(0.83,1.30)	0.720	6464	0.158
	J	1.08	(0.86,1.37)	0.507	6464	
	T	1.00	(0.78,1.28)	0.995	6464	
	K	0.78	(0.58,1.04)	0.093	6464	
	Other European	1.31	(0.99,1.73)	0.056	6464	

^1^ Only girls in the analysis. ^2^ Natural logarithm of DHEAS levels. ^3^ Adjusted for gestational age, sex and 10 principal components. DHEAS: Dehydroepiandrosterone; BMD: Total bone mineral density; YP: Young person; DEXA: dual-energy X-ray absorptiometry.

## Data Availability

Data used for this submission can be accessed after submitting an application to and receiving approval from the ALSPAC Executive (alspac-exec@bristol.ac.uk).

## Supplementary Materials

The following are available online at http://www.mdpi.com/2073-4425/9/8/395/s1, Table S1: Breast cancer risk factors in ALSPAC mothers of European ancestry, Table S2: Breast cancer risk factors in ALSPAC children of European ancestry, Table S3: Distribution of major mitochondrial DNA haplogroups and subgroups in ALSPAC mothers and children of European descent based on HaploGrep quality scores, Table S4: Breast cancer risk factors and major mtDNA haplogroups in ALSPAC mothers of European ancestry, unadjusted analysis, Table S5: Breast cancer risk factors and major mtDNA haplogroups in ALSPAC children of European ancestry, unadjusted analysis, Table S6: *p*-values obtained in the adjusted analysis using a quality score threshold of >90% compared to >80%, Figure S1: Top two principal components (PCs) by mtDNA haplogroup in ALSPAC mothers, Figure S2: Top two principal components (PCs) by mtDNA haplogroup in ALSPAC children.
